# Perioperative Acetaminophen and Dexmedetomidine Eliminate Post-Operative Opioid Requirement following Pediatric Tonsillectomy

**DOI:** 10.3390/jcm11030561

**Published:** 2022-01-23

**Authors:** Andrew G. Rudikoff, David D. Tieu, Franklin M. Banzali, Carolyn V. Nguyen, Robert L. Rettig, Marlene M. Nashed, Janet Mora-Marquez, Qiaoling Chen, Antonio Hernandez Conte, Keira P. Mason

**Affiliations:** 1 Department of Anesthesiology, Kaiser Permanente Los Angeles Medical Center, 4867 Sunset Blvd., Los Angeles, CA 90027, USA; Andrew.G.Rudikoff@kp.org (A.G.R.); Franklin.M.Banzali@kp.org (F.M.B.); 2 Department of Head & Neck Surgery, Kaiser Permanente Los Angeles Medical Center, 4867 Sunset Blvd., Los Angeles, CA 90027, USA; David.D.Tieu@kp.org (D.D.T.); Carolyn.V.Nguyen@kp.org (C.V.N.); 3 Department of Surgery, Kaiser Permanente Los Angeles Medical Center, 4867 Sunset Blvd., Los Angeles, CA 90027, USA; Robert.L.Rettig@kp.org; 4 Department of Pharmacy, Kaiser Permanente Los Angeles Medical Center, 4867 Sunset Blvd., Los Angeles, CA 90027, USA; marlene.m.nashed@kp.org; 5 Department of Research & Evaluation, Kaiser Permanente Southern California, 100 S. Los Robles Avenue, Pasadena, CA 91101, USA; Janet.Mora-Marquez@kp.org (J.M.-M.); Qiaoling.Chen@kp.org (Q.C.); 6 Department of Anesthesiology, Critical Care and Pain Medicine, Boston Children’s Hospital, 300 Longwood Avenue, Boston, MA 02115, USA; keira.mason@childrens.harvard.edu

**Keywords:** tonsillectomy, adenoidectomy, opioid-sparing multimodal analgesia, enhanced recovery after surgery, dexmedetomidine, acetaminophen, fentanyl

## Abstract

Administration of post-operative opioids following pediatric tonsillectomy can elicit respiratory events in this patient population that often arise as central and obstructive sleep apnea. The primary objective of this study was to determine whether a perioperative combination of dexmedetomidine and acetaminophen could eliminate post-operative (in recovery and at home) opioid requirements. Following IRB approval and a waiver for informed consent, the medical records of 681 patients who underwent tonsillectomy between 1 January 2013 and 31 December 2018 were evaluated. Between 1 January 2013 and 31 December 2015, all patients received a fentanyl-sevoflurane-based anesthetic, without acetaminophen or dexmedetomidine, and received opioids in recovery and for discharge home. On 1 January 2016, an institution-wide practice change replaced this protocol with a multimodal perioperative regimen of acetaminophen (intravenous or enteral) and dexmedetomidine and eliminated post-operative opioids. This is the first time that the effect of an acetaminophen and dexmedetomidine combination on the perioperative and home opioid requirement has been reported. Primarily, we compared the need for rescue opioids in the post-anesthesia care period and after discharge. The multi-modal protocol eliminated the need for post-tonsillectomy opioid administration. Dexmedetomidine in combination with acetaminophen eliminated the need for post-operative opioids in the recovery period.

## 1. Introduction

Tonsillectomy is one of the most common pediatric surgical procedures performed in the United States, with an estimated frequency of 289,000 per year [1]. Obstructive sleep apnea (OSA) resulting from adenotonsillar hypertrophy is the most common indication for this operation. Pain control with opiates in the post-operative period (both immediate and following discharge home) is an important area of concern, balancing the analgesic needs with the risk of obstructive sleep apnea, hypopnea and respiratory depression [2]. Recent concerns in opioid-naïve adolescent and adult surgical patients relate to a risk of chronic opiate use in up to 8% of those who are exposed to perioperative opiates [3,4]. Perioperative regimens of oral dextromethorphan and acetaminophen with intraoperative boluses of dexmedetomidine and ketamine have also been successful in a small pilot study of how to eliminate opiates in these tonsillectomy patients [5].

By limiting the opioid-ordering capability and encouraging non-opioid alternative medications on electronic order sets, hospitals have decreased physician opiate orders for post-tonsillectomy children below seven years of age [6]. Other centers have sought to reduce or eliminate opioid use via medication substitution with an opioid alternative such as dexmedetomidine, ketamine, acetaminophen or ibuprofen [7,8,9]. The combination of dexmedetomidine and ketorolac has been shown to be equally effective to morphine and acetaminophen for post-tonsillectomy analgesia [10]. Multimodal approaches using dexmedetomidine, nonsteroidal analgesics and regional anesthesia have been shown to significantly decrease morphine requirements following pediatric tonsillectomy [11].

In 2016, our integrated healthcare system was committed to implementing a comprehensive multimodal analgesic regimen to reduce or eliminate the administration of opioids to pediatric patients undergoing tonsillectomy. The opioid-sparing regimen, using a multimodal approach, had not been previously reported or trialed in the literature and consisted of perioperative dexmedetomidine in combination with acetaminophen. This study reports the outcomes prior to and following this institution-wide mandated change of practice in anesthesia delivery and opiate prescribing patterns.

We hypothesized that the implementation of this opiate-sparing multimodal regimen would decrease perioperative opioid administration and eliminate the need for an opioid prescription at the time of discharge home or the use of opioids while at home.

## 2. Materials and Methods

The study was approved by our Institutional Review Board and patient consent was waived. This study conducted a retrospective review of an existing patient electronic medical record database from 1 January 2013 to 31 December 2018.

### 2.1. Subject Selection

International Classification of Diseases-9 (ICD) and ICD-10 codes were utilized to identify patients who underwent tonsillectomy for obstructive sleep apnea at the Kaiser Permanente Los Angeles Medical Center.

The opiate-based group consisted of patients who underwent tonsillectomy from 1 January 2013 to 31 December 2016 with unrestricted opioid delivery access, and the opiate sparing (multimodal) group from 1 January 2016 to 31 December 2018 represented patients who received perioperative acetaminophen (intravenous or enteral), intravenous dexmedetomidine, and intravenous fentanyl. The two groups were subdivided into cohorts based on age: (1) 0–7 years and (2) 8–13 years.

The cohort comprised pediatric patients (ages 0–14) who had undergone tonsillectomy for a primary indication of obstructive sleep apnea. We excluded patients who received concomitant multiple procedures, such as myringotomies, ear tube insertions, bronchoscopies, direct laryngoscopies and intracapsular tonsillectomy (not considered to be as painful as tonsillectomy).

The primary outcomes were intraoperative morphine administration, post-anesthesia care unit (PACU) opioid administration and the need for home opioids after discharge. Secondary outcome measures included PACU pain scores and readmissions to the emergency room for intractable pain.

### 2.2. Anesthetic Regimen and Surgical Technique

All patients in both the opiate and opiate-sparing/multimodal groups received pre-operative anxiolysis with oral (0.5–0.7 mg/kg) or intravenous midazolam (0.1 mg/kg to a maximum of 2 mg). Anesthesia was initiated via either sevoflurane mask induction or with intravenous propofol, and anesthesia was maintained with sevoflurane following intubation. All patients received dexamethasone (0.5 mg/kg IV; maximum dose 10 mg) IV and ondansetron (0.1 mg/kg; maximum 4 mg) IV at the end of the procedure.

All patients in the opiate group received intraoperative fentanyl (2 mcg/kg) IV. The opiate-sparing group received only 1 mcg/kg IV fentanyl with the multimodal regimen of intravenous dexmedetomidine (1 mcg/kg) and 10–15 mg acetaminophen (oral route preoperatively or intravenous route following induction).

The surgical technique consisted of standard electrocautery for tonsillectomies. Adenoidectomies were performed using the microdebrider technique with suction cauterization. Hemostasis was achieved with suction cauterization.

### 2.3. Post-Operative Pain Management in Post Anesthesia Care Unit

Pain was assessed using a FLACC score in the 0–7 age group. In the 8–13 age group, pain was assessed by a visual analog scale (VAS). Patients in the opiate group were prescribed enteral hydrocodone/acetaminophen (7.5/325 elixir 0.1 mg/kg/dose) every 4–6 h as needed to treat their pain. The multimodal group was prescribed oral oxycodone (0.1 mg/kg), acetaminophen (10 mg/kg) and ibuprofen (10 mg/kg), alternating as needed every 6 h. Patients were instructed that the oxycodone should be limited to breakthrough pain not controlled by acetaminophen and ibuprofen.

### 2.4. Statistical Analysis

The baseline demographics and clinical and surgical characteristics of the study groups were analyzed by the opiate-sparing analgesic regimen status and we subdivided the cohort into two age groups (age 0 to 7 and age 8 to 13). For each group, we calculated the means with standard deviation (SD) or medians with interquartile range (Q1, Q3) for continuous variables and the number of observations with percentages for categorical variables. *p*-values for continuous variables were evaluated with a t-test or Wilcoxon rank-sum test, and a chi-squared test or Fisher’s exact test was utilized for categorical variables for all comparisons between the opiate and opiate-sparing group.

Univariate analyses were stratified by age groups and conducted for different outcome measures. The primary outcome measure of the percentage of patients with opioid use and the morphine milligram equivalent (MME) were compared using a stacked bar plot and box plot, respectively. Secondary outcomes were presented and tested using a Wilcoxon rank-sum test, Chi-squared test or Fisher’s exact test. To evaluate the associations between opiate use in the opiate-sparing group, multivariable logistic regression was performed adjusting for age, sex, race/ethnicity, body mass index (BMI), surgery type and surgical duration. Adjusted odds ratios with corresponding 95% confidence intervals were reported. Multivariable logistic regression was also conducted for secondary outcomes given a significant univariate association. All analyses were performed using SAS statistical software version 9.4 (SAS Institute Inc., Cary, NC, USA).

## 3. Results

### Demographics and Primary Outcome Measures

Table 1 summarizes the demographic information, clinical and surgical characteristics of the study groups, outpatient opioids dispensed and emergency department readmission rates for intractable pain. There were no significant differences between the groups with regard to age, race and BMI.

In the 0–7 age group, significantly fewer opioids were dispensed in the multimodal dexmedetomidine group than in the opiate group (*p* < 0.001); however, there was no difference between the two groups for the 8–13 age group. (*p* = 0.24). There were no significant differences between the control and MMAR groups in the readmissions for any reason or for hemorrhage. In Table 2, all patients in the opiate-sparing group are reported as having less pain than the opiate group on arrival to the PACU. In both age groups, the majority of the patients in the multimodal group were sleeping on arrival to the PACU, while in the opiate group, there was comparatively more mild pain (*p*-value 0.001–0.003). Though there was a reduction in opioid use in the opiate-sparing multimodal group, there were no differences in the pain scores in the PACU for patients who required rescue opioids. Table 2 summarizes the degree of pain the patients had before and after receiving rescue opioids in the PACU between the opiate and opiate-sparing multimodal analgesia groups; there was no significant difference in the degree of pain between these groups.

**Figure 1:**
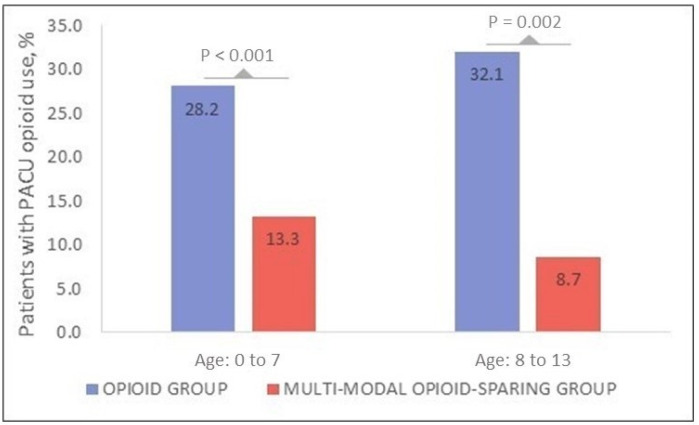
Stacked bar plot graph demonstrating patients who required rescue opioid administration in the post-anesthesia care unit.

Figure 1 is a stacked bar plot that graphically highlights that the opioid consumption and opiate administration in the PACU were significantly lower in the multimodal cohort for both age groups (age 0–7, *p* < 0.001; age 8–13, *p* = 0.002).

## 4. Discussion

This is the first report in the literature that describes a decrease in perioperative narcotic requirement and home opiate intake in children following tonsillectomy with the perioperative dexmedetomidine and acetaminophen combination. Our study is unique because it follows the opioid intake of these children through all three phases of care: intraoperative, post-anesthesia care and at home. Our study’s combination of perioperative acetaminophen and dexmedetomidine with a home regimen of alternating doses of ibuprofen and acetaminophen successfully lowered the intraoperative fentanyl requirement and opioid intake in the PACU and at home.

Prior studies have evaluated solo and combination regimens of dexmedetomidine, bupivicaine, ketamine, magnesium sulfate, ketorolac and acetaminophen in pediatric patients undergoing tonsillectomy. Acetaminophen is one of the most widely studied drugs for adjuvant pain control in tonsillectomy patients. Acetaminophen has been shown to be cost-effective and efficacious compared to opioids alone in this patient population [12,13,14]. However, use of acetaminophen alone compared to dexamethasone alone in tonsillectomy patients was not found to yield improved pain outcomes [12]. Recent studies evaluating the administration of peritonsillar bupivacaine compared to acetaminophen showed promising results for managing post-operative pain [15,16]. Dexmedetomidine has been shown to increase the time to the first need for analgesic medications, as well as prevent emergence agitation and decrease the opioid requirements of patients with OSA undergoing tonsillectomy [9,17,18,19,20]. A meta-analysis demonstrated that intraoperative use of dexmedetomidine was as effective as opioids in preventing post-operative pain and emergence agitation in children who had undergone tonsillectomy and adenoidectomy [21].

In our study, patients receiving multimodal analgesic therapy demonstrated a decreased need for opioids in the PACU, even when adjusted for age, sex, BMI and surgical duration. In the 0–7 years multimodal analgesic group, there was reduced opioid prescription utilization when the patient was discharged home; however, this difference was not observed in the older 8–13 years group. There was no difference in the readmission rates for pain management between the multimodal analgesic and opiate groups. In the <7 years age group, the perioperative combination of acetaminophen and dexmedetomidine, combined with a post-operative ibuprofen and acetaminophen regimen, demonstrates an increased PACU comfort level and decreased opiate consumption in the PACU and at home.

There were several limitations in this study. First, this was a retrospective analysis and not a randomized control trial. However, because all eligible patients were included and the groups were sequentially separated by two different time periods, and because the data collected are part of the electronic medical records, the retrospective limitations were minimized. Another limitation in this study was that the multimodal analgesia group received either oral acetaminophen or IV acetaminophen, and therefore, bioavailability or bioequivalence may have altered our outcomes. Additionally, the time interval for acetaminophen administration was “pre-incision” rather than a discrete time period; this may possibly have altered the time of maximum effect. Nonetheless, patients in the multimodal analgesia group demonstrated a statistically significant difference in opiate consumption and comfort.

In summary, this study demonstrates that dexmedetomidine in combination with acetaminophen eliminates the need for post-operative opioids in the post-anesthesia care unit and at home during the recovery period. Prospective randomized controlled studies with larger groups of patients are necessary to evaluate the dexmedetomidine and acetaminophen perioperative regimen for opiate requirements, post-operative adverse events and possible long-term opiate abuse patterns.

## Figures and Tables

**Table 1:** Patient demographics and surgical characteristics.

	Age 0 to 7			Age 8 to 13		
	Opiate Group (*n* = 354)	Multimodal Analgesia Group (*n* = 150)	*p*-Value	Opiate Group (*n* = 131)	Multimodal Analgesia Group (*n* = 46)	*p*-Value
Age ^1^, mean (SD)	4.3 (1.68)	4.3 (1.86)	0.51	9.9 (1.53)	10.2 (1.66)	0.20
Gender ^2^, *n* (%)			0.86			**0.05**
Female	141 (39.8%)	61 (40.7%)		58 (44.3%)	28 (60.9%)	
Male	213 (60.2%)	89 (59.3%)		73 (55.7%)	18 (39.1%)	
BMI ^1^, mean (SD)	17.2 (3.45)	17.0 (3.16)	0.63	23.4 (6.06)	23.5 (7.00)	0.97
Surgery ^3^, *n* (%)			0.32			>0.99
Tonsillectomy	4 (1.1%)	0 (0%)		7 (5.3%)	2 (4.3%)	
Tonsillectomy with adenoidectomy	350 (98.9%)	150 (100%)		124 (94.7%)	44 (95.7%)	
Anesthesia duration ^4^ (min), median (Q1, Q3)	64.0 (58.0, 73.0)	67.5 (59.0, 75.0)	**0.05**	66.0 (60.0, 75.0)	70.5 (61.0, 81.0)	0.09

*p*-values were generated from ^1^*t*-test or ^4^ Wilcoxon rank-sum test for continuous variables and ^2^ Chi-squared test or ^3^ Fisher’s exact test for categorical variables. Significant results are in bold text.

**Table 2:** Descriptive statistics of secondary study outcomes.

	Age 0 to 7			Age 8 to 13		
	Opiate Group (*n* = 354)	Multimodal Analgesia Group (*n* = 150)	*p*-Value	Opiate Group (*n* = 131)	Multimodal Analgesia Group (*n* = 46)	*p*-Value
PACU duration ^1^ (min), median (Q1, Q3)	95.0 (71.0, 135.0)	108.5 (77.0, 138.0)	0.23	95.0 (69.0, 121.0)	90.0 (70.0, 128.0)	0.78
Outpatient opioid prescribed ^2^, *n* (%)	175 (49.4%)	19 (12.7%)	**<0.001**	69 (52.7%)	24 (52.2%)	0.95
Outpatient opioid consumption ^2^, *n* (%)	90 (25.4%)	10 (6.7%)	**<0.001**	39 (29.8%)	18 (39.1%)	0.24
Readmission for pain ^3^ (overall), *n* (%)	17 (4.8%)	7 (4.7%)	>0.99	12 (9.2%)	6 (13%)	0.57
Pain score on arrival to PACU ^3^, *n* (%)			**0.001**			**0.003**
Sleeping	149 (44.3%)	88 (59.5%)		58 (45.3%)	34 (73.9%)	
Mild pain	166 (49.4%)	55 (37.2%)		61 (47.7%)	11 (23.9%)	
Moderate/severe pain	21 (6.3%)	5 (3.4%)		9 (7%)	1 (2.2%)	

*p*-values were generated from ^1^ Wilcoxon rank-sum test for continuous variables and ^2^ Chi-squared test or ^3^ Fisher’s exact test for categorical variables. Of note, Obese patients were mandatorily admitted after surgery and had a longer length-of-stay. Statistically significant results are in bold.

## Data Availability

Data is maintained with the Kaiser Permanente Regional Research Committee.
